# Differential Effects of Human Adenovirus E1A Protein Isoforms on Aerobic Glycolysis in A549 Human Lung Epithelial Cells

**DOI:** 10.3390/v12060610

**Published:** 2020-06-03

**Authors:** Martin A. Prusinkiewicz, Jessie Tu, Mackenzie J. Dodge, Katelyn M. MacNeil, Sandi Radko-Juettner, Gregory J. Fonseca, Peter Pelka, Joe S. Mymryk

**Affiliations:** 1 Department of Microbiology and Immunology, The University of Western Ontario, London, ON N6A 3K7, Canada; mprusink@uwo.ca (M.A.P.); jtu46@uwo.ca (J.T.); mdodge@uwo.ca (M.J.D.); kmacne9@uwo.ca (K.M.M.); 2 Department of Microbiology, University of Manitoba, Winnipeg, MB R3T 2N2, Canada; sandi.radko@stjude.org (S.R.-J.); peter.pelka@umanitoba.ca (P.P.); 3 Department of Medicine, Division of Quantitative Life Sciences, Meakins-Christie Laboratories, McGill University Health Centre, Montreal, QC H4A 3J1, Canada; gregory.fonseca@mcgill.ca; 4 Department of Medical Microbiology, University of Manitoba, Winnipeg, MB R3T 2N2, Canada; 5 Department of Otolaryngology, Head and Neck Surgery, The University of Western Ontario, London, ON N6A 3K7, Canada; 6 Department of Oncology, The University of Western Ontario, London, ON N6A 3K7, Canada; 7 London Regional Cancer Program, Lawson Health Research Institute, London, ON N6C 2R5, Canada

**Keywords:** glycolysis, cellular respiration, E1A, human adenovirus, 13S, 12S, Warburg effect, oxidative phosphorylation, pentose phosphate pathway, tricarboxylic acid cycle

## Abstract

Viruses alter a multitude of host-cell processes to create a more optimal environment for viral replication. This includes altering metabolism to provide adequate substrates and energy required for replication. Typically, viral infections induce a metabolic phenotype resembling the Warburg effect, with an upregulation of glycolysis and a concurrent decrease in cellular respiration. Human adenovirus (HAdV) has been observed to induce the Warburg effect, which can be partially attributed to the adenovirus protein early region 4, open reading frame 1 (E4orf1). E4orf1 regulates a multitude of host-cell processes to benefit viral replication and can influence cellular metabolism through the transcription factor avian myelocytomatosis viral oncogene homolog (MYC). However, E4orf1 does not explain the full extent of Warburg-like HAdV metabolic reprogramming, especially the accompanying decrease in cellular respiration. The HAdV protein early region 1A (E1A) also modulates the function of the infected cell to promote viral replication. E1A can interact with a wide variety of host-cell proteins, some of which have been shown to interact with metabolic enzymes independently of an interaction with E1A. To determine if the HAdV E1A proteins are responsible for reprogramming cell metabolism, we measured the extracellular acidification rate and oxygen consumption rate of A549 human lung epithelial cells with constitutive endogenous expression of either of the two major E1A isoforms. This was followed by the characterization of transcript levels for genes involved in glycolysis and cellular respiration, and related metabolic pathways. Cells expressing the 13S encoded E1A isoform had drastically increased baseline glycolysis and lower maximal cellular respiration than cells expressing the 12S encoded E1A isoform. Cells expressing the 13S encoded E1A isoform exhibited upregulated expression of glycolysis genes and downregulated expression of cellular respiration genes. However, tricarboxylic acid cycle genes were upregulated, resembling anaplerotic metabolism employed by certain cancers. Upregulation of glycolysis and tricarboxylic acid cycle genes was also apparent in IMR-90 human primary lung fibroblast cells infected with a HAdV-5 mutant virus that expressed the 13S, but not the 12S encoded E1A isoform. In conclusion, it appears that the two major isoforms of E1A differentially influence cellular glycolysis and oxidative phosphorylation and this is at least partially due to the altered regulation of mRNA expression for the genes in these pathways.

## 1. Introduction

Viruses are obligate intracellular parasites as they are only capable of replicating within the infected host cell. Viruses co-opt a wide variety of host cell pathways to meet the requirements for replication. This includes reprogramming cellular metabolism to provide the substrates and energy required for successful replication [1]. Typically, this metabolic reprogramming involves an upregulation of glycolysis and a downregulation of cellular respiration despite the presence of ample oxygen [1]. This is known as aerobic glycolysis. Interestingly, this metabolic phenotype was first observed in cancer cells by Otto Heinrich Warburg in the 1920s [2], and is also referred to as the Warburg effect. For this reason, it is possible there are similarities between the reprogramming of metabolism by viruses and cancer cells. Aside from aerobic glycolysis, viruses can modify a wide range of metabolic pathways including nucleotide biosynthesis, glutamine metabolism, and lipid metabolism [3]. The mechanisms by which different viruses enact these metabolic changes can be specific to the virus [4]. For example, viruses with larger genomes, such as herpes simplex virus 1, may encode some of their own metabolic enzymes [4]. However, the extent to which metabolism can be directly altered by natively encoded viral metabolic proteins is still limited, and virtually non-existent for small viruses, including human adenovirus (HAdV) [5]. Consequently, viruses must enact metabolic changes indirectly by altering the regulation and function of host-cell enzymes and pathways. Typically, this is achieved by a viral protein that acts as a molecular hub. These proteins are capable of regulating the function of other host-cell regulatory proteins, which in turn affect a multitude of cellular processes, including metabolism.

HAdV can dysregulate metabolism [5], potentially through the HAdV early proteins which can enact broad regulatory changes in the host cell [6]. Currently, only the HAdV protein E4orf1 has been implicated in cellular metabolic reprogramming during HAdV infection by causing an increase in glycolysis and glutaminolysis through regulation of the cellular transcription factor MYC [7,8]. While the effects of E4orf1 on cellular metabolism appear to be its most well characterized functions, E4orf1 can also act as an oncoprotein [9], interact with other host-cell proteins through a PDZ domain [10], and potentially contribute to the lytic lifecycle of adenovirus [11]. However, E4orf1 cannot explain the entirety of the metabolic phenomena associated with HAdV infection, including the downregulation of cellular respiration that is noted to occur during infection [8]. Little is known about how the other HAdV oncoproteins regulate metabolism. HAdV E1A is an attractive candidate for contributing to HAdV metabolic reprogramming because it is a viral molecular hub protein [12]. This means that E1A is capable of influencing a wide range of host-cell proteins that regulate various cellular functions, and potentially includes cellular metabolism [13]. Interestingly, this regulation may occur at the transcriptional level due to the ability of E1A to interact with a wide variety of transcription factors that can in turn influence the expression of transcripts encoding enzymes in metabolic pathways (reviewed in [5]). In addition, E1A is the first temporally expressed HAdV protein, which means it could be responsible for influencing early-infection metabolic changes [14,15]. In HAdV-5, the primary E1A transcript is differentially spliced into five isoforms. The two isoforms of E1A encoded by the 13S and 12S mRNAs, are the most predominantly expressed during early infection and are responsible for the majority of its functions [16,17]. They differ only in the presence or absence of one conserved region, known as conserved region 3 (CR3) [18]. However, there are functional differences between these two isoforms, which could contribute to differences in how each influence cellular metabolism. For example, the 13S isoform appears to function more readily as an activator of transcription [19,20], while the 12S isoform regulates cellular activity through transcriptional repression [20] or by influencing localization of host-cell proteins [21]. However, both isoforms are important for productive adenovirus infection and contribute to activating cellular processes related to immortalization and transformation [22]. The purpose of this study was to determine whether the HAdV E1A protein is capable of enacting cellular metabolic changes on its own. To test this, we measured the functional glycolytic and oxidative phosphorylation rates with extracellular flux assays and determined whether they corresponded to changes in the expression of transcripts encoding metabolic genes in A549 cells that stably and endogenously expressed either the 13S or 12S encoded isoforms of E1A. We corroborated these data with RNAseq data from IMR-90 lung fibroblasts infected with mutant variants of HAdV-5 that expressed either the 13S or 12S encoded isoform of E1A. As these isoforms differ only in whether they contain the CR3 region, any differences in metabolism or transcript expression between these two cell lines or viral infections could be ascribed to this region.

## 2. Materials and Methods

### 2.1. Cell Culture

Low passage (<20) A549 cells with endogenous expression of the 13S encoded E1A isoform of HAdV-5 (A549-13S), the 12S encoded isoform of HAdV-5 E1A (A549-12S) or empty vector transduced control cells (A549-EV) were cultured in Dulbecco’s modified Eagle’s medium (DMEM) with 4.5 g/L glucose, L-glutamine, sodium pyruvate, and phenol red (Wisent Inc, Saint-Jean-Baptiste, QC, Canada) supplemented with 10% fetal bovine serum (Wisent Inc), and 1% penicillin-streptomycin (Wisent Inc). The A549-13S cells were previously described in Soriano et al. [23] as A549-E1A289R cells. These cells and the A549-12S and A549-EV cells were generated concurrently as the A549-13S cells and in a similar manner. This involved the use of a LNSX retrovirus vector (described in [24]) with either 12S or 13S E1A cDNA inserted, while the A549-EV cells have the LNSX empty vector inserted. A pool of transduced cells was used to avoid clonal variation. Cells were passaged every 2 to 3 days and split at 70%–80% confluency. Unless otherwise indicated, cells were plated in the above media for all experiments.

### 2.2. Protein Extraction and Western Blot

Protein extraction from A549-13S, A549-12S, and A549-EV cells and subsequent western blots were performed as described previously [25]. The primary antibody used was a mixture of two E1A-specific mouse monoclonal antibodies, M37 and M58 [26]. The blot was imaged using a ChemiDoc XRS+ (Bio-Rad, Hercules, CA, USA).

### 2.3. Seahorse Glycolytic Stress Test

A549-13S, A549-12S and A549-EV cells were seeded on XFe24 microplates (Agilent, Santa Clara, CA, USA) at a density of 1 × 10^5^ cells/mL for 24 h. Following this, cells were washed in D-PBS (Wisent Inc) and incubated in Seahorse XF base medium (DMEM-based with no bicarbonate, glucose, or pyruvate; Agilent) supplemented with 2 mM L-glutamine at 37 °C in a CO_2_-free incubator for 60 min. The microplate was transferred to a Seahorse XFe24 Analyzer (Agilent) to measure the extracellular acidification rate (ECAR). Basal ECAR was recorded for 3 measurement cycles before injection of glycolytic stress test compounds. Measurements were taken during sequential cycles of exposure to 10 mM glucose (3 measurement cycles), 1.5 μg/mL oligomycin (3 measurement cycles), and 50 mM 2-deoxyglucose (2-DG) (3 measurement cycles). The ECAR was normalized to the third measurement prior to injection of the first compound.

### 2.4. Seahorse Mitochondrial Stress Test

Cells were seeded on XFe24 microplates as above, however the Seahorse XF base medium was supplemented with 2 mM L-glutamine, 10 mM glucose, and 2 mM sodium pyruvate. The microplate was transferred to a Seahorse XFe24 Analyzer to measure the oxygen consumption rate (OCR). Basal OCR was recorded for 3 measurement cycles before injection of mitochondrial stress test compounds. Measurements were taken during sequential cycles of exposure to 1.5 μg/mL oligomycin (3 measurement cycles), 1 μM carbonyl cyanide-4-(trifluoromethoxy)phenylhydrazone (FCCP) (3 measurement cycles), and 0.5 μM rotenone/antimycin A (3 measurement cycles). The OCR was normalized to the third measurement prior to injection of the first compound.

### 2.5. RNA Extraction and qPCR

A549-13S, A549-12S, and A549-EV cells were grown on 100 mm cell culture dishes until 70%–80% confluency. Cells were trypsinized and collected in a cell pellet. Total RNA was extracted with a PureLink RNA mini kit (Invitrogen, Waltham, MA, USA) according to the manufacturer’s guidelines. A total of 1 μg of total RNA was used in a reverse transcription reaction with a SuperScript VILO cDNA synthesis kit (Invitrogen) according to the manufacturer’s guidelines. Primer sequences were generated *de novo* using Primer-BLAST [27] with requirements that the primer pair span an exon-exon junction and be separated by at least one intron when possible. All primer efficiencies were verified using a five-point standard curve with 400 ng, 200 ng, 100 ng, 50 ng and 25 ng of cDNA. A list of primer sequences used in this study can be found in Supplementary Table S1. A total of 50 ng of cDNA per reaction was used for subsequent qPCR characterization of mRNA expression. All qPCR reactions were performed on a QuantStudio 5 Real-Time PCR system (Applied Biosystems, Foster City, CA, USA). *H2AFY* and *ACTB* were used as reference genes. Data were analyzed using the 2^−ΔΔCT^ method.

### 2.6. RNA Sequencing Analysis

IMR-90 primary lung fibroblasts (American Type Culture Collection, Manassas, VA, USA) were contact arrested for 72-h and infected for 16 h with either a *dl520* HAdV-5 mutant [28] (from S.T. Bayley, McMaster University, Hamilton, ON, Canada), which does not express the 12S encoded E1A isoform; a *pm975* HAdV-5 mutant [29] (from S.T. Bayley), which does not express the 13S encoded E1A isoform; or an E1A-deleted HAdV-5 mutant control at a multiplicity of infection of 10. The control virus has the E1 region replaced with CMV-driven beta-galactosidase. Total RNA from infected IMR-90 cells were collected with TRIzol reagent (Sigma, St. Louis, MO, USA) according to the manufacturer’s protocol, with each infection repeated for a total of two biological replicates. Collected RNA was sent to Genome Quebec for processing and sequencing using Illumina’s HiSeq platform. Bam sequencing files were aligned to the hg38 (human) genome using STAR [30]. Tag directories were produced using the homer [31] function makeTagDirectories and RNA reads were quantified using analyzeRepeats. Differential expression was calculated using DESeq2 [32] at a cutoff *p*-value of 0.05.

### 2.7. Statistics

Normalized Seahorse XFe24 Analyzer data were analyzed in GraphPad Prism 8 (San Diego, CA, USA) using a two-way ANOVA in which the ECAR or OCR was defined as the continuous dependent variable, cell line was one categorical independent variable, and injection was the second categorical independent variable. The two-way ANOVA was followed by a Tukey’s multiple comparison test in which each cell mean was compared to every other cell mean on that row. A separate analysis was performed for the glycolytic stress test data and mitochondrial stress test data. qPCR data were analyzed using Microsoft Excel 365 (Redmond, WA, USA) and comparisons between groups were performed using a Student’s t-test.

## 3. Results

### 3.1. A549-13S Cells Increased Baseline Glycolysis and Decreased Maximum Respiration

**Figure 1:**
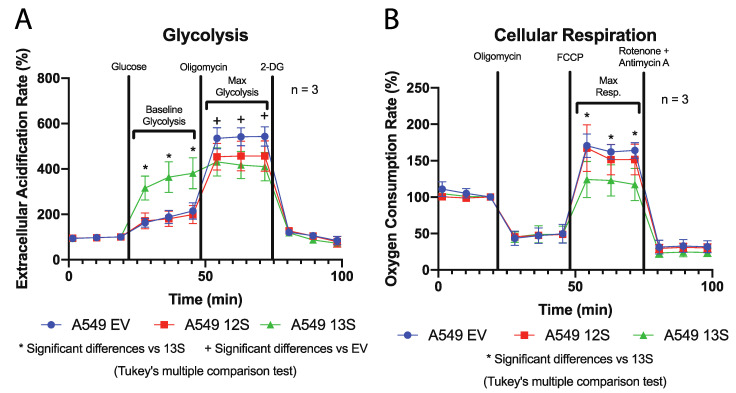
A549-13S cells have a unique functional glycolytic and oxidative phosphorylation metabolism when compared to A549-12S and A549-EV cells. (**A**) Seahorse XFe24 assay of extracellular acidification rates, a readout of glycolysis. Extracellular acidification rates were highest in A549-13S cells after addition of glucose, which is a reflection of the baseline glycolytic rate after stimulation. However, A549-EV cells had the highest maximum extracellular acidification rates after addition of oligomycin, which forces maximal glycolysis by inhibition of ATP synthase. There were no differences in response after the addition of 2-deoxyglucose (2-DG) to shut down glycolysis and end the experiment. * = *p* < 0.05 in a comparison between A549-13S and either A549-12S or A549-EV cell lines. + = *p* < 0.05 in a comparison between A549-EV and either A549-12S or A549-13S cell lines. (**B**) Seahorse XFe24 assay of oxygen consumption rates, a readout of oxidative phosphorylation. The amount of cellular respiration dedicated to ATP production was no different between the cell lines as indicated by oligomycin treatment. Maximal oxygen consumption rates, induced by carbonyl cyanide-4-(trifluoromethoxy)phenylhydrazone (FCCP) which decouples the mitochondria, were lowest in A549-13S cells. There were also no differences between the cell lines after the addition of rotenone and antimycin A used to terminate the experiment. * = *p* < 0.05 in a comparison between A549-13S and either A549-12S or A549-EV cell lines. 2-DG, 2-deoxyglucose; FCCP, carbonyl cyanide-4-(trifluoromethoxy)phenylhydrazone.

To examine the role of adenovirus E1A in changing cellular metabolism, we examined extracellular metabolic flux, with a Seahorse XFe24 analyzer, in A549 cells expressing either the 13S encoded HAdV-5 E1A isoform (A549-13S), the 12S encoded isoform (A549-12S), or an empty vector control (A549-EV). The expression of E1A in these cell lines was confirmed by western blot (Supplementary Figure S1). The extracellular acidification rate of these three cell lines, a readout of glycolytic function, is shown in Figure 1A. The A549-EV and A549-12S cell lines had very similar glycolytic profiles. The minimal basal rate of glycolysis, induced by glucose after two hours of serum starvation, was similar between the two cell lines. To determine their maximal glycolytic potential, cells were stimulated with oligomycin. Oligomycin inhibits ATP synthase, forcing cells to increase glycolysis to a maximal rate. Maximal glycolysis was significantly higher in A549-EV cells than in A549-12S cells. In contrast, A549-13S cells reached their maximal glycolytic rate immediately after the addition of glucose, which implied that the baseline rate of glycolysis in A549-13S cells was higher than in A549-12S cells or A549-EV cells. Importantly, glycolysis in A549-13S cells could not be increased to a higher maximal rate with the addition of oligomycin. In fact, the rate of glycolysis after oligomycin addition was the lowest in A549-13S cells in comparison to both the A549-12S and A549-EV cells. All cell lines responded similarly to 2-DG, which was used to shut down glycolysis and terminate the experiment.

We next assessed oxygen consumption rates, a readout of cellular respiration. The only observed differences in oxygen consumption were in the maximal respiration rates (Figure 1B). Maximal respiration was induced by FCCP, which uncouples oxidation from phosphorylation in mitochondria rendering them inefficient. This causes the cell to increase its rate of cellular respiration to compensate for the mitochondrial inefficiency. The A549-13S containing cells had a lower rate of cellular respiration compared to the A549-12S containing cells or the A549-EV containing cells, which were otherwise equivalent. No differences in response between the cell lines were observed when they were treated with oligomycin, which is used to determine the amount of cellular respiration dedicated to ATP production. Cells were treated with rotenone and antimycin A to completely shut down cellular respiration and terminate the experiment. There were also no differences in response to rotenone and antimycin A between the cell lines.

### 3.2. Glycolytic Genes Are Upregulated in A59-13S Cells

**Figure 2:**
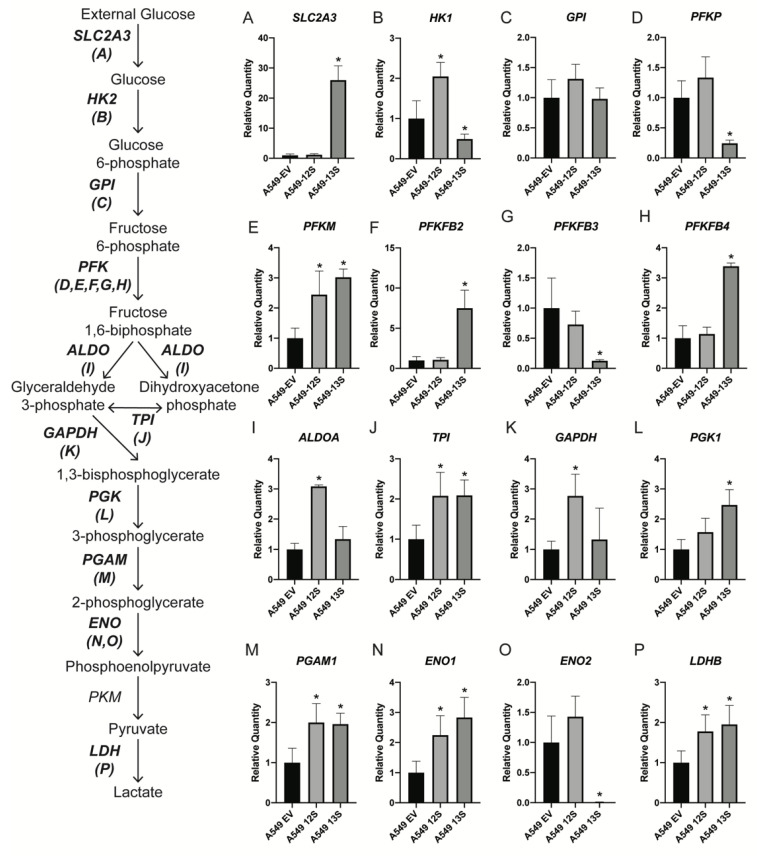
Relative expression levels of mRNA for glycolytic genes in 13S and 12S expressing A549 cells. The mRNA expression for 16 genes encoding components of glycolysis were measured with qPCR (**A**–**P**). A549-13S cells had higher expression of nine genes (**A**,**E**,**F**,**H**,**J**,**L**,**M**,**N**,**P**) and lower expression of four genes (**B**,**D**,**G**,**O**) when compared to A549 cells expressing an empty vector. A549-12S cells had higher expression of eight genes (**B**,**E**,**I**,**J**,**K**,**M**,**N**,**P**) when compared to A549-EV cells. The combination of differential mRNA expression in the A549-13S cells may contribute to their unusual glycolytic phenotype. *H2AFY* was used as a reference gene. Asterisks (*) indicate *p* < 0.05. n = 3 per group for all panels. *SLC2A3*, Solute carrier family 2 member 3; *HK*, hexokinase; *GPI*, Glucose-6-phosphate isomerase; *PFKP*, Phosphofructokinase, platelet; *PFKM*, Phosphofructokinase, muscle; *PFKFB*, 6-phosphofructo-2-kinase/fructose-2,6-biphosphatase; *ALDO*, Aldolase, fructose biphosphate; *TPI*, triosephosphate isomerase; *GAPDH*, Glyceraldehyde 3-phosphate dehydrogenase; *PGK*, Phosphoglycerate kinase; *PGAM1*, Phosphoglycerate mutase; *ENO*, enolase; *PKM*, Pyruvate kinase M1/2; *LDH*, Lactate dehydrogenase.

To determine how these functional differences in metabolism are reflected by the transcription of metabolic genes, we first examined genes involved in glycolysis with qPCR (Figure 2). mRNA for the glucose transporter *SLC2A3* was 26-fold higher in the A549-13S cells than either the A549-12S cells or the A549-EV cells (Figure 2A). Interestingly, hexokinase 1 (*HK1*) mRNA, which encodes the enzyme used in the first step of glycolysis, was significantly lower in A549-13S cells than A549-12S or A549-EV cells (Figure 2B).

There were no differences in mRNA expression of glucose-6-phosphate isomerase (*GPI*) mRNA, which encodes the second enzyme involved in glycolysis, across the three cell lines (Figure 2C). Expression of mRNA encoding phosphofructokinase (*PFK*) isoforms, involved in the third step of glycolysis, varied across its five isoforms. *PFKP* and *PFKFB3* mRNAs were significantly lower in A549-13S cells than either A549-EV or A549-12S cells (Figure 2D,G). *PFKM*, *PFKFB2* and *PFKFB4* mRNAs were significantly higher in A549-13S cells when compared to A549-EV cells (Figure 2E,F,H).

Aldolase (*ALDOA*) mRNA, encoding the enzyme involved in the fourth step of glycolysis, was only significantly higher in A549-12S cells compared to the other two cell lines (Figure 2I). Triose-phosphate isomerase (*TPI*) mRNA, which encodes the fifth glycolytic enzyme, was higher in both the A549-13S and A549-12S cells than the A549-EV cells (Figure 2J). Glyceraldehyde 3-phosphate dehydrogenase (*GAPDH*) mRNA, encoding the sixth glycolytic enzyme, was only significantly higher in A549-12S cells (Figure 2K). mRNA for phosphoglycerate kinase 1 (*PGK1*), which is the seventh glycolytic enzyme, was significantly higher in A549-13S cells than either A549-EV or A549-12S cells (Figure 2L). Transcript levels for phosphoglycerate mutase (*PGAM1*), which encodes the eighth enzyme in glycolysis, were equally high in both A549-13S and A549-12S cells when compared to A549-EV cells (Figure 2M). mRNA expression of enolase 1 (*ENO1*), the ninth glycolytic enzyme, was higher in both A549-12S and A549-13S cells when compared to A549-EV (Figure 2N). However, mRNA for another enolase isoform, enolase 2 (*ENO2*) was virtually non-existent in the A549-13S containing cells, while the amount of *ENO2* mRNA was not significantly different between A549-EV and A549-12S containing cells (Figure 2O). mRNA expression of lactate dehydrogenase B (*LDHB*) which encodes the enzyme responsible for the conversion of pyruvate to lactate in aerobic glycolysis was identified to be significantly higher in A549-13S and A549-12S cells when compared to A549-EV cells (Figure 2P).

### 3.3. Pentose Phosphate Pathway Genes Are Differentially Regulated in A549-13S Cells

**Figure 3:**
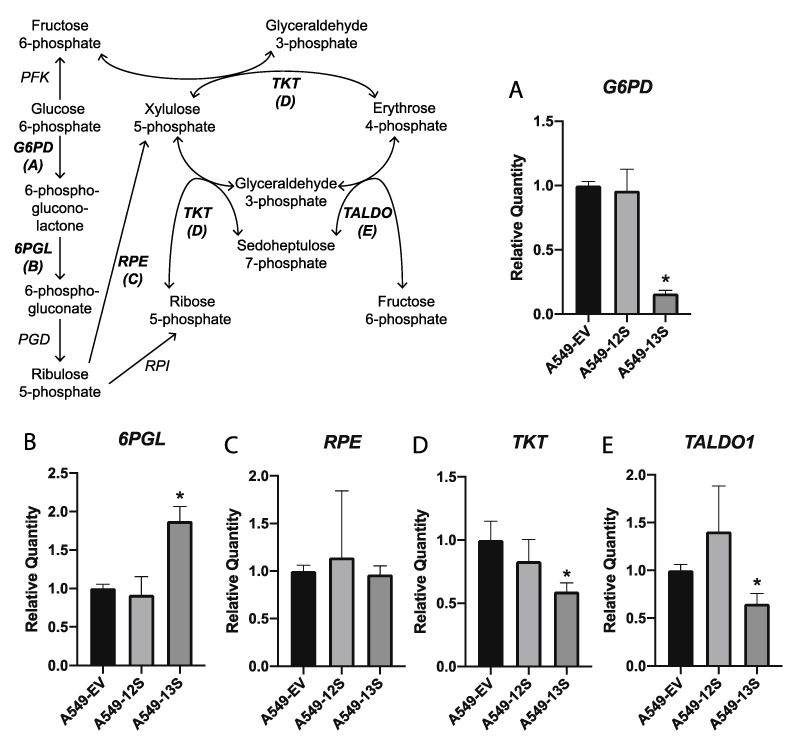
Relative expression of mRNA for pentose phosphate pathway genes in 13S and 12S expressing A549 cells. The mRNA expression for five genes encoding components of the pentose phosphate pathway were measured with qPCR (**A**–**E**). The pentose phosphate pathway relies on the products of glycolysis. Only the A549-13S cells displayed differential regulation of transcripts in this pathway. Of the five pentose phosphate pathway genes measured, three were downregulated (**A**,**D**,**E**) and one was upregulated in the A549-13S cells (**B**). *H2AFY* was used as a reference gene. Asterisks (*) indicate *p* < 0.05. n = three per group for all panels. *G6PD*, Glucose-6-phosphate dehydrogenase; *6PGL*, 6-phosphogluconolactonase; *PGD*, Phosphogluconate dehydrogenase; *RPE*, Ribulose-5-phosphate-3-epimerase; *RPI*, Ribose 5-phosphate isomerase; *TKT*, Transketolase; *TALDO*, Transaldolase; *PFK*, Phosphofructokinase.

The mRNA expression levels of five genes involved in the pentose phosphate pathway (PPP) were also characterized in these three A549 derived cell lines. mRNA of glucose-6-phosphate dehydrogenase (*G6PD*), which encodes the first enzyme of the PPP oxidative branch, was significantly lower in the A549-13S cells than either the A549-EV or A549-12S cells (Figure 3A). However, mRNA for 6-phosphogluconolaconase (*6PGL*), which encodes the second enzyme of the PPP oxidative branch, was significantly higher in A549-13S cells (Figure 3B).

The mRNA expression levels of ribulose-5-phosphate-3-epimerase (*RPE*), which encodes the enzymatic link between the oxidative and non-oxidative branches of the PPP, was not significantly different across the three cell lines (Figure 3C). mRNA transcripts for transketolase (*TKT*) and transaldolase 1 (*TALDO1*), which encode the two enzymes involved in the non-oxidative branch of the PPP, were significantly lower in A549-13S cells, but not in A549-12S cells or A549-EV cells (Figure 3D,E).

### 3.4. Tricarboxylic Acid Cycle Genes Are Upregulated in A549-13S Cells

**Figure 4:**
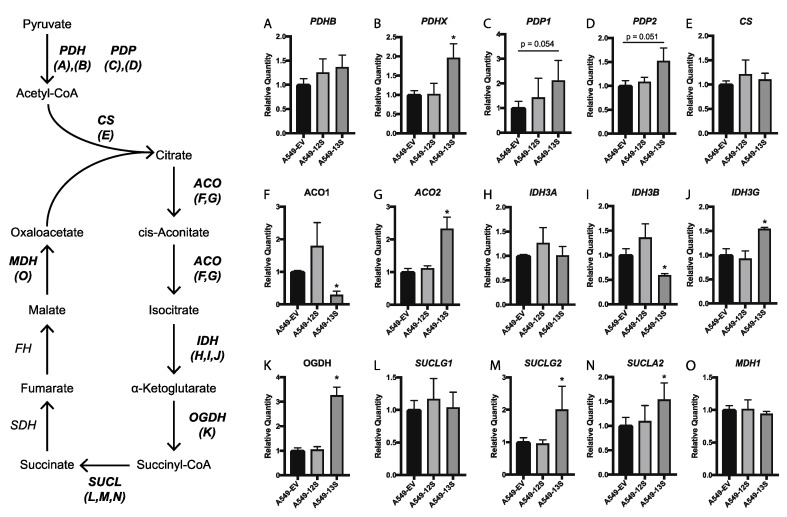
Relative expression of mRNA for tricarboxylic acid cycle genes in 13S and 12S expressing A549 cells. (**A**–**O**) The mRNA expression for 15 genes encoding components of the tricarboxylic acid (TCA) cycle were measured with qPCR. A549-13S cells displayed upregulated mRNA levels of enzymes involved in the TCA cycle. A549-13S cells had statistically significant upregulation of transcripts encoding six enzymes involved in the TCA cycle (**B**,**G**,**J**,**K**,**M**,**N**) and downregulation of two TCA cycle enzyme encoding transcripts (**F**,**I**). In contrast, A549-12S cells did not exhibit any statistically significant differences in TCA cycle transcript expression compared to the A549-EV cells. The geometric mean of two reference genes, *H2AFY* and *ACTB* was used. Asterisks (*) indicate *p* < 0.05. n = three per group for all panels. *PDH*, pyruvate dehydrogenase; *PDP*, Pyruvate dehydrogenase phosphatase; *CS*, Citrate synthase; *ACO*, Aconitase; *IDH*, Isocitrate dehydrogenase; *OGDH*, Oxoglutarate dehydrogenase; *SUCLG1*, Succinate-CoA ligase GDP/ADP-forming subunit alpha; *SUCLG2*, Succinate-CoA ligase GDP-forming subunit beta; *SDH*, Succinate dehydrogenase; *FH*, Fumarate hydratase; *MDH*, Malate dehydrogenase.

The upregulation of many glycoytic genes by E1A, including *LDHB*, suggested that A549-13S cells were preferentially utilizing aerobic glycolysis. However, this does not preclude an upregulation of the tricarboxylic acid (TCA) cycle. For example, many cancer cells and virally infected cells upregulate select enzymes involved in the TCA cycle to promote glutaminolysis and other anaplerotic reactions [33,34,35]. Indeed, when we measured the expression of 15 genes involved in the TCA cycle with qPCR, six were upregulated only in A549-13S cells (Figure 4).

The first was pyruvate dehydrogenase complex component x (*PDHX*) mRNA, which encodes a component of the pyruvate dehydrogenase complex that is responsible for converting pyruvate to acetyl-CoA (Figure 4B). The next upregulated transcript was aconitase 2 (*ACO2*) mRNA, which encodes an enzyme responsible for the second step of the TCA cycle. In this step the metabolite citrate is converted into isocitrate through the intermediate cis-aconitate. Expression of *ACO2* was expressed over 2-fold more in A549-13S cells than A549-EV cells (Figure 4G). The third upregulated transcript is isocitrate dehydrogenase (NAD(+)) 3 non-catalytic subunit gamma (*IDH3G*) mRNA, which encodes a subunit of the isocitrate dehydrogenase complex. This complex is responsible for the third step of the TCA cycle, the conversion of isocitrate into α-ketolgutarate. *IDH3* was upregulated 1.5-fold compared to the A549-EV (Figure 4J). The following upregulated TCA cycle transcript was oxoglutarate dehydrogenase (*OGDH*) mRNA. This gene encodes a component of the 2-oxoglutarate dehydrogenase complex, which is responsible for the conversion of α-ketoglutarate into succinyl-CoA. *OGDH* expression was three-fold higher in A549-13S cells than A549-EV cells (Figure 4K). The final two upregulated TCA cycle transcripts in A549-13S cells encode components of succinyl-CoA synthetase, which is responsible for the conversion of succinyl-CoA to succinate. These transcripts were succinate-CoA ligase GDP-forming subunit beta (*SUCLG2*) mRNA and succinate-CoA ligase ADP-forming subunit beta (*SUCLA2*) mRNA. *SUCLG2* expression was two-fold higher (Figure 4M) and *SUCLA2* expression was 1.5-fold higher (Figure 4N) in A549-13S cells than A549-EV cells. Interestingly, there were no significant differences in TCA cycle gene expression between the A549-12S cells and the A549-EV cells.

### 3.5. Oxidative Phosphorylation Genes Are Downregulated in A549-13S Cells

**Figure 5:**
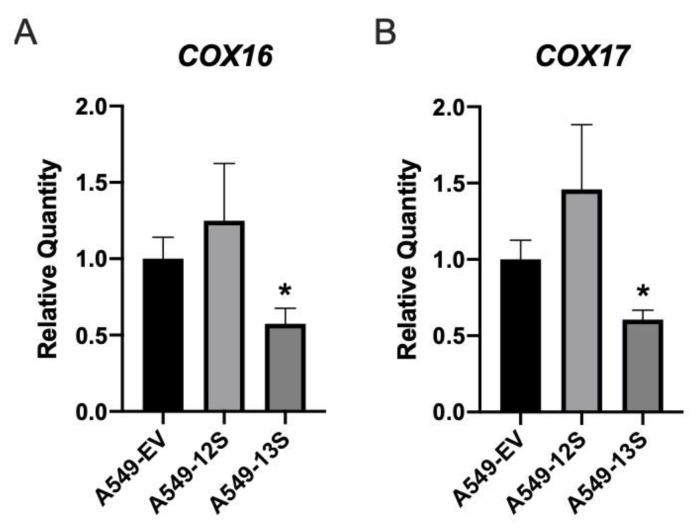
Relative expression of mRNA for cellular respiration genes in 13S and 12S expressing A549 cells. The mRNA expression for two genes encoding components of the TCA cycle were measured with qPCR. A549-13S cells exhibit downregulated expression of genes encoding components of cellular respiration complex IV. Both (**A**) *COX16* and (**B**) *COX17* mRNA expression was significantly lower in A549-13S cells compared to A549-EV cells. A549-12S cells did not show statistically significant expression differences when compared to A549-12S cells. The geometric mean of two reference genes, *H2AFY* and *ACTB* was used. Asterisks (*) indicate *p* < 0.05. n = three per group for all panels. *COX16*; Cytochrome C oxidase assembly factor COX16; *COX17*, Cytochrome C oxidase copper chaperone COX17.

An upregulation of genes involved in the TCA cycle may allow for increased glutaminolysis or anaplerotic pathways rather than impacting cellular respiration. This would likely be reflected in a decrease in the expression of cellular respiration genes, despite the upregulation of TCA cycle genes. We determined the expression levels of two transcripts that encoded components of cellular respiration complex IV, cytochrome C oxidase assembly factor COX16 (*COX16*) mRNA (Figure 5A) and cytochrome C oxidase copper chaperone COX17 (*COX17*) mRNA (Figure 5B). The expression of both *COX16* and *COX17* was significantly lower in A549-13S cells. This paralleled the decreased oxygen consumption rate observed in A549-13S cells (Figure 1B). There were no differences in the expression of these two genes in A549-12S cells when compared to A549-EV cells.

### 3.6. 13S E1A Influences Metabolism to a Greater Extent than 12S E1A in HAdV-5 Infected Primary IMR-90 Cells

To determine the physiological relevance of the 13S encoded E1A isoform on cellular metabolic reprogramming in the context of infection, in contrast to the 12S encoded isoform, IMR-90 primary lung fibroblast cells were grown to confluence for 72 h and infected with mutant HAdV-5 expressing either the 13S or 12S E1A isoforms. Cells were infected with either the *pm975* mutant of HAdV-5, which predominantly expresses the 13S, but not the 12S encoded E1A isoform; the *dl520* HAdV-5 mutant, which predominantly expresses the 12S, but not the 13S isoform; or an E1A-deleted HAdV-5 mutant as a control. RNA from these cells was collected at 16 h post infection, which corresponds to a relatively early timepoint during infection, at which E1A should be influencing cellular gene expression [17]. The expression of RNA from these cells was determined using RNAseq.

**Figure 6:**
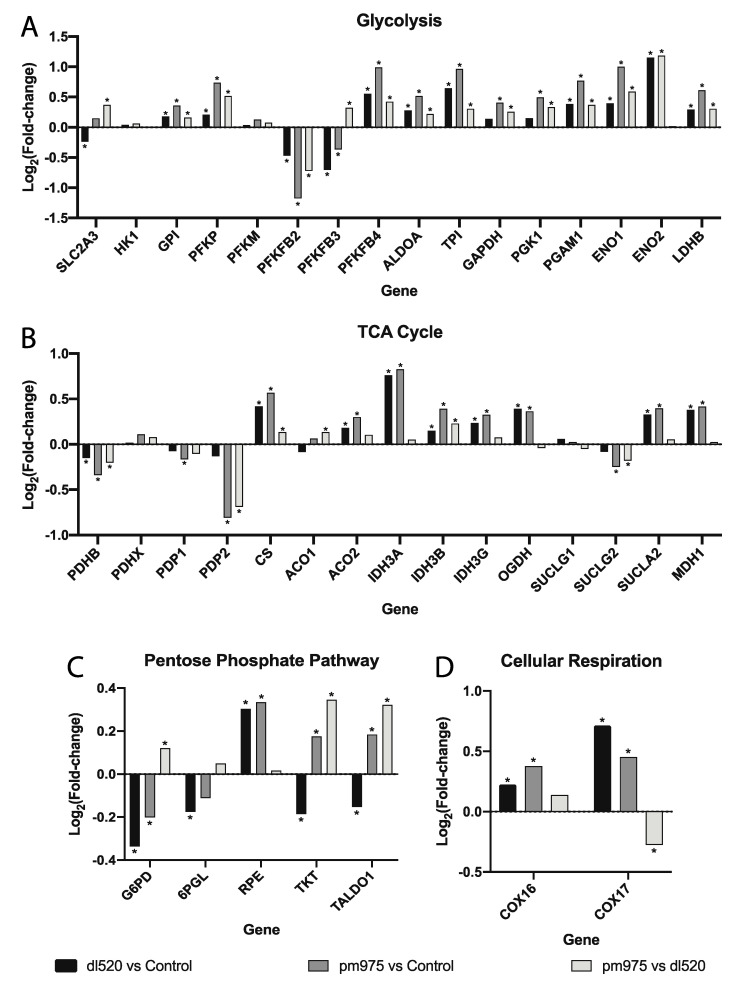
RNAseq analysis of metabolic genes from IMR-90 lung fibroblasts infected with either *dl520*, *pm975* or an E1A-deleted HAdV-5 control virus. (**A**) RNA expression of glycolytic enzyme-encoding transcripts was often greater in *pm975* infected cells than *dl520* infected cells. However, *dl520* infected cells also expressed glycolytic genes at consistently higher levels than in an E1A-deleted control HAdV-5 infection. (**B**) RNA expression of transcripts encoding TCA cycle enzymes, more highly up- or downregulated in *pm975* infected cells when compared to *dl520* infected cells. (**C**) Transcripts encoding pentose phosphate pathway intermediates in the non-oxidative pentose phosphate pathway branch are more highly upregulated in *pm975* infected cells than in *dl520* infected cells. (**D**) Both *COX16* and *COX17* transcripts, which encode components of the TCA cycle, are upregulated in both *pm975* and *dl520* infected cells, although to a lesser extent in the *pm975* infection. Asterisks (*) indicate an adjusted *p*-value < 0.05. n = two per group for all panels. Gene names are defined in Figure 2, Figure 3, Figure 4 and Figure 5.

Expression of the metabolic genes analyzed in the above RT-qPCR experiments was extracted from the RNAseq data allowing for the comparison of *dl520* infected cells versus control virus infected cells, *pm975* infected cells versus control virus infected cells, and *pm975* infected cells versus *dl520* infected cells (Figure 6). In terms of glycolytic mRNA expression (Figure 6A), 10 of the transcripts were more highly upregulated in *pm975* infected cells than in *dl520* infected cells. In agreement with the A549 cell line results presented above (Figure 2), suggesting that the 13S encoded isoform of E1A has a more pronounced role in promoting glycolytic metabolic reprogramming than the 12S encoded isoform during infection. However, both HAdV-5 mutants appear to upregulate glycolytic transcript expression to a greater extent than E1A-deleted HAdV-5.

The expression of transcripts for enzymes in the TCA cycle appear to be less regulated by E1A at 16 h post infection than was observed with the A549 cell lines (compare Figure 4 and Figure 6B). However, there are still some differences in TCA cycle transcript expression between the *pm975* and the *dl520* mutant infections. *PDHB*, *PDP2*, and *SUCLG2* are all more highly downregulated in *pm975* infected cells than in *dl520* infected cells, while *CS*, and *IDH3B* are more highly upregulated. *ACO2*, *IDH3A*, *IDH3G*, *OGDH*, *SUCLA2*, and *MDH1* have higher levels of transcript expression in both *pm975* and *dl520* infected cells when compared to E1A-deleted control HAdV-5 infected cells, but the expression of these transcripts was not significantly different between *pm975* and *dl520* infected cells.

Transcripts encoding enzymes involved in the PPP (Figure 6C) appear to be upregulated in *pm975* infected cells as compared to either *dl520* infected cells or the E1A-deleted control. While the expression of *G6PD* is lower in both *pm975* and *dl520* infected cells when compared to the E1A-deleted control HAdV-5 infected cells, *G6PD* is less drastically downregulated in the *pm975* infected cells. In addition, transcripts for PPP enzymes in the non-oxidative branch, *RPE*, *TKT*, and *TALDO1*, were significantly higher in *pm975* infected cells than either *dl520* or E1A-deleted control HAdV-5 infected cells. The expression of these three genes was significantly lower in the *dl520* infected cells when compared to the E1A-deleted HAdV-5 infections.

Finally, for the two cellular respiration related transcripts, expression of *COX16* showed an upregulation in both *dl520* and *pm975* infected cells when compared to E1A-deleted HAdV-5 control infections, but not to each other. Transcript expression of *COX17* was also up in both *dl520* and *pm975* infections, but this upregulation was less drastic in the *pm975* infected cells when compared to *dl520* infected cells.

## 4. Discussion

To determine the role that E1A plays in altering cellular metabolism, we investigated the impact of constitutive expression of either of the two major E1A isoforms on the metabolism of A549 lung epithelial cells. Interestingly, A549-13S cells reach a high maximal level of glycolysis after the addition of glucose, unlike the A549-12S cells or the A549-EV controls. The A549-12S and A549-EV cells require the ATP synthase inhibitor oligomycin to reach a maximum ECAR. Oligomycin has been suggested to promote glycolysis by inducing the translocation of GLUT1 glucose transporters to the plasma membrane [36]. As hypothesized, E1A appears to be at least partially responsible for controlling these changes at a transcriptional level. In our analysis of the mRNA expression for genes involved in glycolysis, *SLC2A3,* which encodes the glucose transporter GLUT3, was 26-fold higher in the A549-13S cells than the A549-12S or A549-EV cells. This suggests that E1A 13S is also capable of inducing glycolysis through upregulation of glucose transporters, which could explain why the addition of oligomycin to further induce glycolysis was ineffective in these cells. The upregulation of *SLC2A3* transcript expression in only the A549-13S cells may explain why these cells exhibit constitutively high levels of glycolysis while the A549-12S cells do not, despite both cells upregulating some glycolysis genes in comparison to the A549-EV cells. An additional indication that A549-13S cells could functionally upregulate glycolysis while the A549-12S cells could not, was that a greater number of glycolytic genes appeared to be upregulated in the A549-13S cells than the A549-12S cells. However, there were glycolytic transcripts uniquely upregulated in the A549-12S cells and not the A549-13S cells, these being *HK1*, *ALDOA*, and *GAPDH*. Conversely, the glycolytic transcripts *HK1*, *PFKP*, *PFKFB3,* and *ENO2* were uniquely downregulated in A549-13S cells. Both of these results suggest that the regulation of glycolysis by E1A may not be solely dependent on direct transcriptional regulation. The products of these transcripts with apparently paradoxical up- or downregulation in relation to the functional glycolytic extracellular flux data could be regulated post-transcriptionally in an appropriate manner. This could include regulation of metabolic enzymes with post-translational modifications, competition between metabolite intermediates, and feedback loops (reviewed in [37,38,39]). Future work exploring how E1A influences metabolism through these mechanisms could reveal the extent that E1A regulates metabolism in ways unrelated to transcription.

Many metabolic pathways use glycolytic intermediates as precursors. For example, the PPP relies on glucose-6-phosphate as a precursor to produce the ribose sugar backbone of nucleotides (Figure 3). However, while *6PGL*, from the oxidative branch of the PPP, was upregulated in the A549-13S cells, expression of *G6PD* from the oxidative branch was downregulated. In addition, the expression of two genes encoding enzymes from the nonoxidative branch of the PPP, *TKT* and *TALDO1*, were both lower. This suggests that E1A 13S inhibits the PPP. Peak nucleotide production occurs at time points that follow peak E1A expression [40]. Perhaps E1A 13S plays a role in modulating the timing of nucleotide production by downregulation of the PPP. It is also interesting to note that none of the A549-12S cells had any statistically significant differences in mRNA expression in the PPP when compared to the A549-EV cells, which may further support the idea that the E1A 12S isoform is not involved in the regulation of metabolism during infection.

The TCA cycle is another metabolic pathway that utilizes glycolytic products to provide biosynthetic intermediates that can be utilized in other pathways. The TCA cycle also pulls electrons from intermediates to later produce energy through the electron transport chain. Interestingly, the TCA cycle gene that had the highest level of expression in the A549-13S cells was *OGDH*, which was three-fold higher than in A549-EV cells. OGDH is a subunit of the α-ketoglutarate dehydrogenase complex, which is responsible for converting α-ketoglutarate into succinyl-CoA. Cells that utilize aspartate for anaplerotic metabolism are particularly reliant on OGDH [41], which suggests that the TCA cycle in A549-13S cells may be utilized for biomolecule synthesis. In addition, the α-ketoglutarate dehydrogenase complex represents a key step of glutaminolysis [42], which again points towards A549-13S cells utilizing the TCA cycle for biosynthetic metabolic reactions rather than the electron transport chain. Two genes, *SUCLG2* and *SUCLA2*, which encode components of succinyl-CoA synthetase, the complex which immediately follows α-ketoglutarate dehydrogenase in the TCA cycle, were also uniquely upregulated in the A549-13S cells. Succinyl-CoA synthetase activity has also been implicated in anaplerotic metabolism [43]. Finally, there is an opposing upregulation of *IDH3G* and downregulation of *IDH3B* in A549-13S cells, both of which encode components of the isocitrate dehydrogenase, the complex immediately preceding the α-ketoglutarate dehydrogenase complex in the TCA cycle. However, *IDH3B* has been suggested to be a hub gene that drives tumour associated cellular pathways [44], and it could be functioning in a similar manner when its expression is potentially driven by E1A 13S. A decreased rate of functional cellular respiration in the A549-13S cells, combined with lower expression of *COX16* and *COX17* in these cells is another indication that the TCA cycle is being reprogrammed for biosynthetic or anaplerotic reactions.

It should also be noted that the A549 parent cell line, used to generate the cell lines expressing either the 13S or 12S encoded isoforms of E1A, is derived from a human epithelial lung carcinoma. This means at baseline it is expected that this cell line would exhibit some aspects of the Warburg effect in relation to a primary cell line from the same tissue. However, as the A549-13S cells exhibited a drastic increase in glycolysis and drastic decrease to cellular respiration when compared to either the A549-12S or A549-EV cell lines, this reveals the existence of overriding effects of the 13S encoded E1A isoform on host-cell metabolic reprogramming. It is possible that if a similar experiment were conducted using transduced lung primary cell lines, an even more drastic upregulation of glycolysis and downregulation of cellular respiration might be observed. While this study shows that the 13S encoded isoform of E1A will likely influence metabolism in primary cells, it does not definitively exclude the possibility that 12S encoded E1A isoform could have lesser effects on reprogramming primary cell metabolism to more closely resemble the Warburg effect.

The question remains as to how these two different E1A isoforms may specifically influence metabolism in the context of adenovirus infection. Considering that HAdV infected cells tend to exhibit an upregulation of glycolysis and a downregulation of oxidative phosphorylation [8,33] similar to the A549-13S cells in this study, it is likely that this isoform contributes to metabolic reprogramming during HAdV infection. The effect of the 13S isoform on metabolism may be especially predominant as expression of the 12S isoform is typically three times higher than the 13S isoform when both are at their peak at approximately 24 h post infection [17].

To explore this question, RNAseq data from IMR-90 cells infected for 16 h were analyzed for expression of the genes used for our qPCR analysis. In terms of glycolysis, the 13S encoded isoform of E1A altered expression of these genes to a greater extent than the 12S encoded isoform. However, a lesser upregulation of glycolysis-related transcript expression was also observed in cells infected with the HAdV-5 mutant that expressed the 12S E1A isoform compared to the control virus infected cells. This suggests that while the 13S isoform likely plays a more significant role in the upregulation of glycolysis during infection, the 12S isoform may still influence glycolysis. While changes in the expression of TCA cycle transcripts did not appear to be greatly different between the *pm975* and *dl520* infected cells, TCA cycle transcripts were still much higher than control virus infected cells. It is possible that the TCA cycle is partially upregulated at 16 h post infection to provide some of the substrates and energy required for viral replication, and this is driven in part by E1A. In addition, some of the upregulated transcripts were unique to *pm975* HAdV-5 infected cells, which matches our qPCR data in which A549 cells expressing the 13S isoform uniquely upregulated a greater number of different TCA cycle genes compared to cells expressing the 12S isoform.

Interestingly, transcripts encoding enzymes in the nonoxidative branch of the PPP were uniquely upregulated in cells infected with *pm975*, again suggesting that the 13S encoded isoform of E1A promotes host-cell metabolic activity during infection. Although this was opposite to the observed downregulation of nonoxidative branch PPP transcripts observed in A549-13S cells, in both cases the 13S encoded isoform of E1A appears to be regulating the PPP. It appears that during *dl520* infection, the 12S isoform is downregulating the nonoxidative branch of the PPP. Finally, and unexpectedly, the two examined transcripts encoding components of cellular respiration, *COX16* and *COX17* were upregulated in both *dl520* and *pm975* infected cells. This could imply that at this early infection timepoint, the infected cell is being driven into a highly metabolically active state to promote viral replication, and cellular respiration may contribute to this process, at least marginally. It would be useful to determine whether the 13S encoded E1A isoform coordinates a downregulation of cellular respiration transcripts at later stages of infection.

In addition, although the smaller isoforms of E1A, such as the 9S, 10S and 11S encoded E1A isoforms become more predominant during later stages of infection [17], it is unclear whether they would contribute to regulation of host-cell metabolism during HAdV infection. Perhaps they could modulate metabolic pathways regulated during later stages of infection, such as nucleotide production, but this remains to be explored.

Understanding how viruses alter host-cell metabolic processes during infection could yield insight into potential antiviral interventions. For example, understanding the parallels between HAdV-5 metabolic reprogramming of infected host cells with that of other viruses, such as influenza [45], could have implications for the universal treatment of viruses with compounds that target similar viral processes during infection. In addition, this study provides more evidence for the outsized role the 13S encoded isoform of E1A could have in deregulating host-cell processes during HAdV infection. As the 13S encoded isoform differs from the 12S encoded isoform of E1A by CR3, this region could represent a unique and targetable weak point of HAdV. For example, the 13S encoded E1A isoform is especially important for productive lytic infection of HAdV in A549 cells, while the 12S encoded isoform is less efficient at promoting HAdV induced lysis [46]. It would be interesting to determine whether simply modulating metabolic pathways upregulated by the 13S encoded isoform would be enough to hinder productive HAdV infection.

## 5. Conclusions

The purpose of this study was to determine whether either of the two major isoforms of HAdV-5 E1A, the 13S or the 12S encoded isoforms, were able to modulate cellular metabolism in cell lines that endogenously expressed these proteins. The difference between the 13S and 12S encoded isoforms is the presence or absence of the CR3 region in the larger major E1A protein [47], which targets and modulates the activity of a distinct set of host-cell regulatory proteins that are primarily involved in transcriptional regulation [48,49]. Given the similarity between these E1A isoforms, differences in metabolic function or the transcription of metabolic genes between these two isoforms is likely related to the presence of this region. Interestingly, we observed that A549-13S cells had robust glycolytic capacity and reduced cellular respiration when compared to A549-12S or A549-EV cells. This corresponded to a unique upregulation of transcripts encoding enzymes involved in glycolysis and a downregulation of transcripts encoding enzymes involved in cellular respiration in the A549-13S cells compared to the other lines. This was also accompanied by a downregulation of PPP transcripts in A549-13S cells, and an upregulation of TCA cycle transcripts consistent with anaplerotic metabolism. This enhanced upregulation of glycolysis was also apparent in primary IMR-90 lung fibroblasts infected with a HAdV-5 mutant virus that expressed the 13S isoform of E1A. E1A is a viral oncoprotein [50], and it is also interesting that the larger E1A isoform can reprogram metabolism in a manner that resembles the Warburg effect, including an upregulation of baseline glycolysis and downregulation of cellular respiration. This work strongly suggests that E1A is an additional HAdV protein capable of influencing cellular metabolism.

## Supplementary Materials

The following are available online at https://www.mdpi.com/1999-4915/12/6/610/s1, Figure S1: Western blot of E1A in A549-EV, A549-12S and A549-13S cell lines, Table S1: Primers List.
