# Supplementary material for: Differential Effects of Human Adenovirus E1A Protein Isoforms on Aerobic Glycolysis in A549 Human Lung Epithelial Cells
Source: Viruses. 2020 Jun 3;12(6):610. doi: 10.3390/v12060610 (PMC7354625; doi:10.3390/v12060610)
Supplement: Supplementary file 1 [file viruses-12-00610-s001.zip › Supplementary Files/2020.04.13 - Supplementary Figure 1.docx]

**Supplementary Figure 1.** Western blot of E1A expression levels in A549-EV, A549-12S and A549-13S cell lines. Replicate cell lysates were resolved by electrophoresis, transferred to membrane and blotted with a mixture of M37 and M58 E1A-specific mouse antibodies. No E1A was detected in lanes 1 and 2, corresponding to the A549-EV cells that do not express E1A. As expected, A549-12S cells (lanes 3-6) transduced with a vector expressing the smaller major isoform of E1A showed a band with a lower molecular weight than A549-13S cells (lanes 7-10) transduced with a vector expressing the larger major isoform of E1A. Note that each consecutive pair of lanes corresponds to protein from a single sample.
